# Dr. Tweedie on a Peculiar Swelling of the Lower Extremity, Post Febrem

**Published:** 1829-01-01

**Authors:** 


### LONDON FEVER HOSPITAL.

#### I. SWELLING OF THE LOWER EXTREMITY, POST FEBREM.

The very talented and observant physician of the London Fever Hospital, (Dr. Tweedie) has published, in our northern contemporary, a short, but interesting paper on a "peculiar swelling of the lower extremity, which occasionally comes on during the progress of, or convalescence from fever." It has been cursorily noticed by Dr. Cheyne, as an affection resembling *phlegmatia dolens*—to which, in Dr. Tweedie's opinion, also, it bears a considerable resemblance, in character and progress.

"This disease, which I conceive to be inflammation of the cellular tissue of the limbs, differs, however, in many particulars. For example, in all the instances I have seen the swelling has been confined to one extremity, making its appearance first about the upper part, extending gradually over the whole limb, and being attended with acute pain. It does not retain the impression of the finger, as in common œdema, which generally commences round the ankle and foot, and seldom extends much higher.

"I have treated several cases of this affection within the last four years at the London Fever Hospital. All the subjects of it were females, two of whom were under twenty years of age, and unmarried. I have been informed, however, that one case occurred in a male; but I have not been able to trace the history or treatment."

In all the cases which came under Dr. Tweedie's care, active depletion had been employed for the fever, so that the convalescence was tedious. The swelling in question was ushered in with symptoms of general excitement, which led the Doctor to expect relapse, or visceral inflammation. In a few hours, however, the local affection was announced by complaint of stiffness in one of the lower extremities, followed by aching pain, either about the upper and inner part of the thigh, or in the ham, around the knee, or calf of the leg. In the course of twelve or eighteen hours, the pain and stiffness increase, and, on the limb being examined, it is found to be somewhat swollen, and perceptibly hotter than the opposite. But there is no redness of the skin, which, on the contrary, has a smooth, white, shining appearance, and the cutaneous veins are distended with blood, and occasionally tortuous. "As the disease advances, the swelling extends uniformly over the limb from the upper part of the thigh to the toes, and feels tense and elastic, but not at all diminished by the semiflexed position of the limb, which the patient generally prefers." Dr. T. also remarked the total inability to move the limb—a peculiarity noticed by Burns, in the puerperal *phlegmatia dolens*.

When active treatment is employed, the pain abates—the swelling loses its elasticity and tension, so as partly to pit on pressure—the heat of the limb diminishes—but the power of motion continues long impaired. In one case, the disease terminated in ill-conditioned suppuration; and fluctuation being discovered, an incision was made, when a considerable quantity of thin sero-purulent fluid escaped. There was no wall or cyst, in this case, to confine the matter, as in common abscess. The patient recovered after a tedious convalescence. Two cases are detailed in illustration of the foregoing descriptions and remarks.

We conceive that Dr. Tweedie's paper tends to illustrate the true pathology of *phlegmatia dolens*—for we cannot see any characteristic or essential difference between that disease as it appears after parturition, and the one here described by the talented author of the paper. That they are *both* a peculiar inflammation, affecting, not merely the inguinal veins, but a variety of structures (more especially perhaps the cellular membrane) of the limb, we think there cannot be a doubt. The paper of Dr. T. is therefore valuable, as it bears on the pathology—and even the *etiology* of another and troublesome disease, respecting which, there has been much discussion of late.

---

### XIII.

### CHELTENHAM CASUALTY HOSPITAL.

I On the Removal of loose Substances from the Knee-joint. By Charles Averill, Esq.

